# P-2030. Streamlining Root Cause Analysis of Healthcare-Associated Infections and Outbreaks Using a Mobile App–Based Digital Platform: A Quality Improvement Study in Infection Prevention and Control

**DOI:** 10.1093/ofid/ofaf695.2194

**Published:** 2026-01-11

**Authors:** Jose J Kochuparambil, Dona Ann Varkey

**Affiliations:** MQMH, Pala, Kerala, India; Mary Queens Mission Hospital, Anakkallu, Kerala, India

## Abstract

**Background:**

Timely Root Cause Analysis (RCA) of healthcare-associated infections (HAIs) is essential for effective infection control. This study evaluated a mobile app–based RCA platform to improve response time, implementation, and recurrence tracking in a tertiary care hospital.

**Methods:**

This prospective, before-and-after quality improvement study was conducted from January to June 2024 in a 650-bed tertiary hospital in South India. The mobile app integrated event-specific RCA templates (CLABSI, CAUTI, SSI, VAP), timeline tracking, task assignment, escalation alerts, and digital signatures. RCA data from July to December 2023 (paper-based) served as controls. Outcomes included median time to RCA closure, corrective action completion rate, event recurrence within 60 days, and user engagement and satisfaction. Statistical analyses included Kaplan-Meier survival curves, Cox proportional hazards regression, and chi-square tests.

**Results:**

A total of 53 RCA events were included (control: 26; intervention: 27). The Kaplan–Meier survival analysis showed a significantly faster time to RCA closure in the intervention group (*log-rank p* < 0.001). Median RCA closure time was reduced from 12.5 days to 4.2 days. Using Cox proportional hazards regression, digital RCA implementation was associated with a 3.08-fold increased likelihood of timely closure (Hazard Ratio: 3.08, 95% CI: 1.85–5.13, *p* < 0.001), after adjusting for infection type, unit, and severity. Subgroup analysis demonstrated the greatest time reduction for CLABSI-related RCAs (median: 14 to 3.5 days, *p* < 0.01). Corrective action implementation rates improved from 57.6% (15/26) in the control group to 88.9% (24/27) in the intervention group (*χ²* = 7.05, *p* = 0.008). The event recurrence rate within 60 days dropped significantly from 19.2% (5/26) to 3.7% (1/27) (*χ²* = 4.89, *p* = 0.026). User satisfaction was high, with a mean System Usability Scale (SUS) score of 85.6 ± 6.3 and a Net Promoter Score (NPS) of +54. Task acceptance within 48 hours improved from 46% to 91% post-implementation (*p* < 0.001).

**Conclusion:**

The mobile RCA platform led to significantly faster investigation cycles, improved accountability, and reduced infection recurrence. This digital framework can transform RCA implementation in IPC programs, especially in LMICs.

**Disclosures:**

All Authors: No reported disclosures

